# A Digital Smartphone-Based Self-administered Tool (R+ Dietitian) for Nutritional Risk Screening and Dietary Assessment in Hospitalized Patients With Cancer: Evaluation and Diagnostic Accuracy Study

**DOI:** 10.2196/40316

**Published:** 2022-10-26

**Authors:** Zhiwen Long, Shan Huang, Jie Zhang, Deng Zhang, Jun Yin, Chengyuan He, Qinqiu Zhang, Huilin Xu, Huimin He, Ho Ching Sun, Ke Xie

**Affiliations:** 1 Recovery Plus Clinic Chengdu China; 2 Department of Oncology, Sichuan Academy of Medical Sciences and Sichuan Provincial People's Hospital Chengdu China; 3 School of Medicine, University of Electronic Science and Technology of China Chengdu China; 4 College of Food Science, Sichuan Agricultural University Ya'an China

**Keywords:** digital tool, nutritional risk screening, dietary assessment, validity, cancer patients

## Abstract

**Background:**

Malnutrition is a common and severe problem in patients with cancer that directly increases the incidence of complications and significantly deteriorates quality of life. Nutritional risk screening and dietary assessment are critical because they are the basis for providing personalized nutritional support. No digital smartphone-based self-administered tool for nutritional risk screening and dietary assessment among hospitalized patients with cancer has been developed and evaluated.

**Objective:**

This study aims to develop a digital smartphone-based self-administered mini program for nutritional risk screening and dietary assessment for hospitalized patients with cancer and to evaluate the validity of the mini program.

**Methods:**

We have developed the R+ Dietitian mini program, which consists of 3 parts: (1) collection of basic information of patients, (2) nutritional risk screening, and (3) dietary energy and protein assessment. The face-to-face paper-based Nutritional Risk Screening (NRS-2002), the Patient-Generated Subjective Global Assessment Short Form (PG-SGA-SF), and 3 days of 24-hour dietary recall (3d-24HRs) questionnaires were administered according to standard procedure by 2 trained dietitians as the reference methods. Sensitivity, specificity, positive predictive value, negative predictive value, κ value, and correlation coefficients (CCs) of nutritional risk screened in R+ Dietitian against the reference methods, as well as the difference and CCs of estimated dietary energy and protein intakes between R+ Dietitian and 3d-24HRs were calculated to evaluate the validity of R+ Dietitian.

**Results:**

A total of 244 hospitalized patients with cancer were recruited to evaluate the validity of R+ Dietitian. The NRS-2002 and PG-SGA-SF tools in R+ Dietitian showed high accuracy, sensitivity, and specificity (77.5%, 81.0%, and 76.7% and 69.3%, 84.5%, and 64.5%, respectively), and fair agreement (κ=0.42 and 0.37, respectively; CC 0.62 and 0.56, respectively) with the NRS-2002 and PG-SGA-SF tools administered by dietitians. The estimated intakes of dietary energy and protein were significantly higher (*P*<.001 for both) in R+ Dietitian (mean difference of energy intake: 144.2 kcal, SD 454.8; median difference of protein intake: 10.7 g, IQR 9.5-39.8), and showed fair agreement (CC 0.59 and 0.47, respectively), compared with 3d-24HRs performed by dietitians.

**Conclusions:**

The identified nutritional risk and assessment of dietary intakes of energy and protein in R+ Dietitian displayed a fair agreement with the screening and assessment conducted by dietitians. R+ Dietitian has the potential to be a tool for nutritional risk screening and dietary intake assessment among hospitalized patients with cancer.

**Trial Registration:**

Chinese Clinical Trial Registry ChiCTR1900026324; https://www.chictr.org.cn/showprojen.aspx?proj=41528

## Introduction

Cancer has become a primary public health problem in China over the past several decades [1]. Malnutrition is a common and severe problem in patients with cancer that directly increases the incidence of complications and significantly deteriorates their quality of life [2,3]. Hospitalized malnourished patients with cancer comprise 30%-80% of all patients with cancer [4]. Nausea, vomiting, and diarrhea induced by chemotherapy and radiotherapy can aggravate the nutritional status of patients with cancer [5,6], but nutritional support can improve their clinical outcomes [7-9]. Nutritional risk screening and dietary assessment are critical because they are the basis for providing personalized nutritional support [10].

Guidelines from the Chinese Society for Parenteral and Enteral Nutrition (CSPEN) [11], the American Society for Parenteral and Enteral Nutrition (ASPEN) [12], and the European Society for Clinical Nutrition and Metabolism (ESPEN) [13] recommended that hospitalized patients with cancer should be screened for nutritional risk at admission to enable timely recognition and treatment of nutritional derangements. For those who are classified as having nutritional risk, further objective and quantitative assessment of nutritional intake should be undertaken, especially their energy and protein intakes, which are 2 of the most important nutrients for patients with cancer [14-16].

However, it is difficult to screen all hospitalized patients for nutritional risk in hospitals in China, not to mention assessment of nutritional intake, which is more complex. On the one hand, there is a shortage of nutritional specialists in hospitals in China. Even some tertiary hospitals, which usually employ most medical specialists and provide full component of medical services, do not have departments of nutrition. On the other hand, clinicians and nurses are often under pressure to perform a variety of tasks. Nutritional risk screening and dietary assessment can significantly increase the burden of clinicians and nurses and so they do not perform these tasks routinely. In the coming years, there will still be a shortage of nutritional specialists in hospitals in China, meaning clinicians and nurses will continue to work in a busy clinical environment, but importantly patients’ need for nutritional care should not be neglected. Thus, there is an extremely urgent need for more effective, less time-consuming, and less people-demanding tools.

Modern advancements in digital technologies provide a feasible solution for this problem. For example, a computer-based electronic version of the Malnutrition Universal Screening Tool (MUST), which is used for outpatient self-screening, displayed high validity for nutritional risk self-screening. A dietary assessment app (MyFood) showed good ability to estimate dietary intake [17] and another electronic system improved the documentation of nutritional intake, treatment, and nutritional care plans in hospitalized patients [18]. Several other digital tools based on computers or smartphones were also proved to enhance the efficacy in identifying patients at nutritional risk and assessing dietary intake [19-24]. However, to the best of our knowledge, no study has been conducted on developing a digital smartphone-based self-administered tool for nutritional risk screening and dietary assessment for hospitalized patients with cancer.

We developed a mini program, R+ Dietitian, for smartphones as a support system for nutritional risk screening and dietary assessment for hospitalized patients with cancer. It is a self-administered tool and requires patients to input data about their disease, weight, dietary intake, among other variables. Further nutritional assessment and individualized nutritional care plan would be customized based on the data entered. Therefore, this mini program should be validated to ensure clinicians can provide appropriate nutritional care for patients.

The aims of this study were (1) to develop a digital smartphone-based self-administered mini program for nutritional risk screening and dietary assessment of hospitalized patients with cancer, (2) to evaluate the validity of the mini program for patients’ self-screening nutritional risk compared with dietitians’ professional screening, and (3) to evaluate the validity of the mini program for estimating dietary energy and protein intakes compared with dietitians’ professional estimation using 3 days of 24-hour dietary recalls (3d-24HRs).

## Methods

### Development of the R+ Dietitian Mini Program

#### Overview

R+ Dietitian was developed by dietitians, developers, and interaction designers at Recovery Plus Inc. (R+). Clinicians and nurses at the Oncology Department of Sichuan People’s Hospital were involved in the literary design process.

The initial paper draft, including content and algorithm, was designed by dietitians at R+. Clinicians and nurses at the Oncology Department of Sichuan People’s Hospital reviewed the draft and then gave feedback to dietitians at R+. The content and language of the mini program were then modified before starting the technical development. Next, the beta version of R+ Dietitian was developed and then tested by 4 dietitians, 2 nurses, and 10 patients with cancer. Their feedback was used for further modification prior to commencing this study.

Given the popularity of WeChat, which is China’s most popular messaging app with a monthly user base of more than 1 billion people [25], we developed R+ Dietitian as a subapp within the WeChat ecosystem. This was intended to save users’ time, as this avoids the need to additionally download a new app on their cell phones. Instead, users just need to open their WeChat profile to run R+ Dietitian quickly and smoothly.

All contents displayed in R+ Dietitian were in Chinese. R+ Dietitian consists of the following 3 parts.

#### Part 1: Collection of Basic Information of Patients

**Figure 1:**
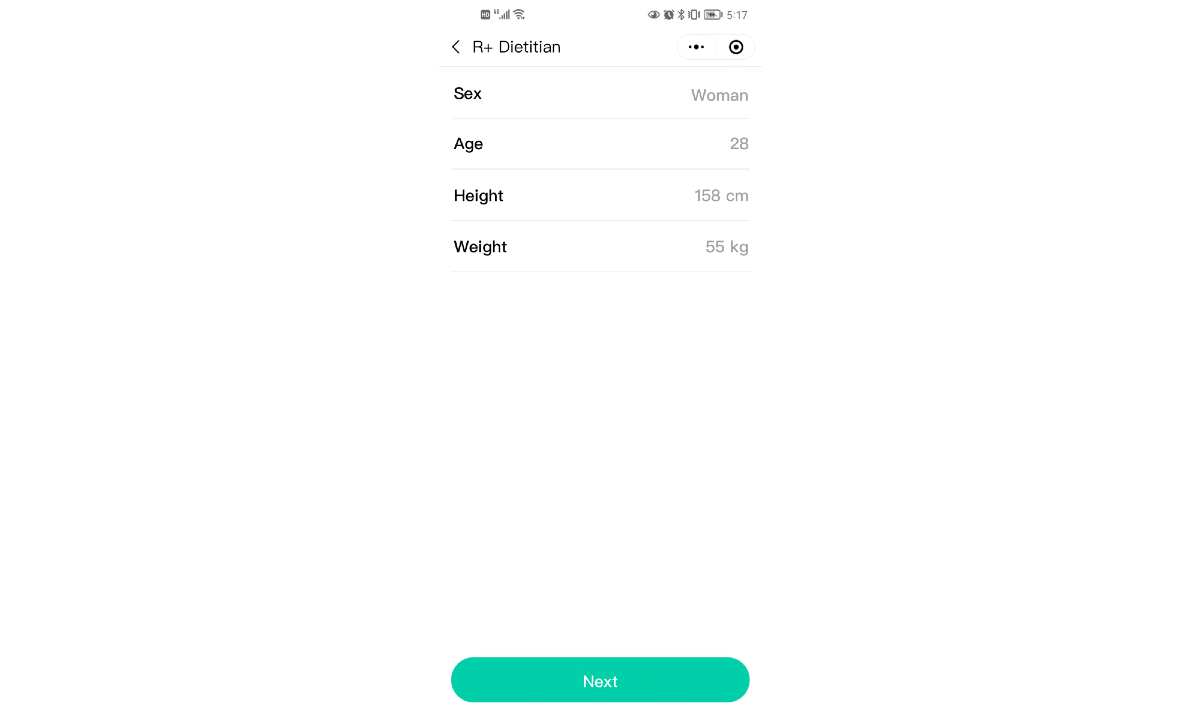
Collection of basic information of patients.

In the first 2 interfaces of R+ Dietitian (Figure 1), patients recorded their basic information, including name, inpatient admission number, bed number, age (in years), gender, height (in centimeters), and weight (in kilograms).

#### Part 2: Nutritional Risk Screening

##### Design

This part was designed based on the Nutritional Risk Screening (NRS-2002) tool and the Patient-Generated Subjective Global Assessment Short Form (PG-SGA-SF) tool. The NRS-2002 tool is recommended by the CSPEN to screen for nutritional risk in the hospital settings [11], whereas the PG-SGA-SF tool is usually used to screen for nutritional risk in patients with cancer [26-28]. Therefore, the estimated NRS-2002 and PG-SGA-SF scores would be given to participants automatically and separately, while their nutritional risk was separately assessed based on these 2 scores.

##### Design and Nutritional Risk Score Estimation of NRS-2002 in R+ Dietitian

The NRS-2002 tool includes 2 components, the initial screening and the final screening. The initial screening has 4 questions. If the answer is “Yes” to any question on the initial screening, the final screening is performed [29]. In China, the initial screening is skipped and only the final screening is performed according to the standard practice [30]. Therefore, the NRS-2002 in R+ Dietitian was aligned with the final screening of the NRS-2002, which consists of 3 sections: impaired nutritional status (scores 0-3), severity of the disease (scores 0-3), and age (scores 0-1), with a total score of 7. Age was already recorded by patients in part 1.

Severity of the disease was evaluated by the question “Please choose the disease you have (multiple choice)” with a disease list covering all types of cancer, common chronic disease, and other diseases that are displayed in the NRS-2002.

**Figure 2:**
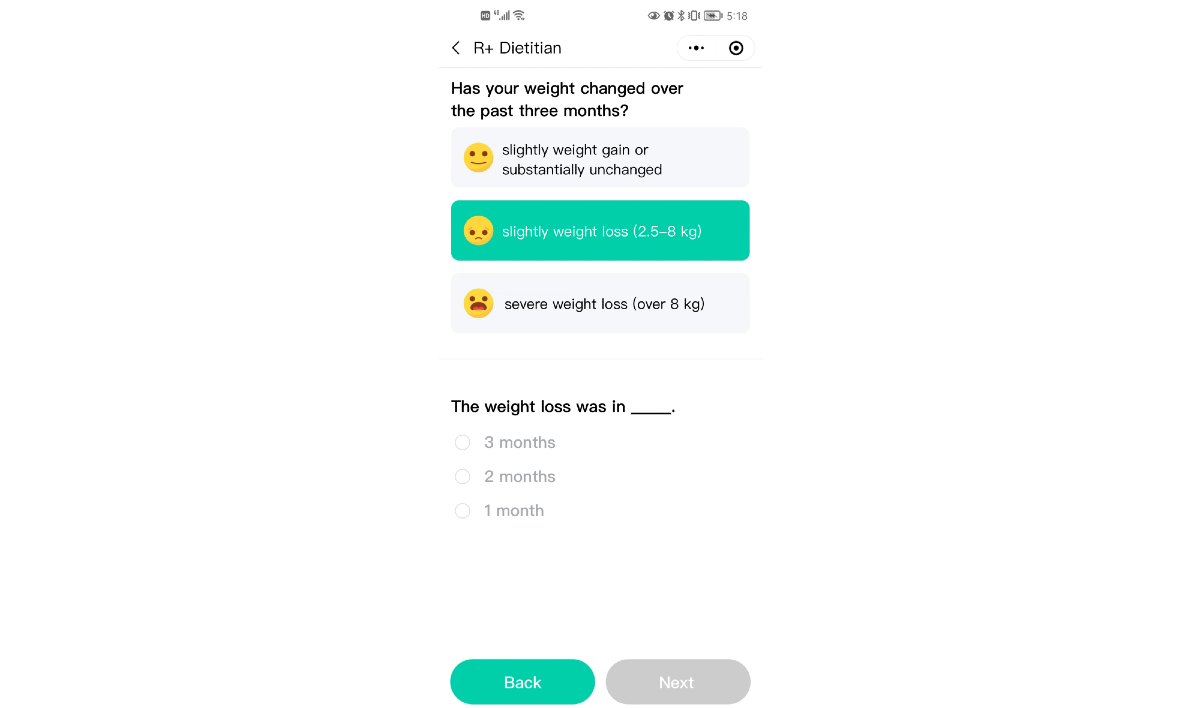
Evaluation for degree of weight loss.

Impaired nutritional status involved 3 indicators: degree of weight loss, BMI, and degree of food intake reduction. BMI of patients was calculated as patients’ weight in kilogram (kg) divided by the square of height in meters (m). Degree of weight loss was evaluated by the question (Figure 2) “Has your weight changed over the past three months?” with 3 options, “slight weight gain or substantially unchanged (weight loss less than 5%),” “slight weight loss (weight loss of 5%-15%),” and “severe weight loss (weight loss exceeding 15%).” The exact values of 5% and 15% weight loss would be calculated automatically through the algorithm set in the back end of R+ Dietitian based on the weight recorded in part 1. The calculated weight loss will be displayed within parentheses next to each option so that patients could clearly choose the right option. For example, if a patient recorded the weight as 58 kg, then options such as “slight weight gain or substantially unchanged (weight loss less than 5 kg),” “slight weight loss (weight loss of 5-17 kg),” and “severe weight loss (weight loss exceeding 17 kg).” would be presented. If patients chose 1 of the latter 2 options, an additional question was presented, namely, “Weight loss in” with 3 options, “3 months,” “2 months,” and “1 month.” Degree of food intake reduction was evaluated by the question “Has there been a reduction in your food intake recently?” with 4 options, “substantially unchanged,” “slight reduction (reduction of about 25%-50%),” “moderate reduction (reduction of about 50%-75%),” “severe reduction (reduction of about 75%-100%).”

Based on age, severity of the disease, and impaired nutritional status reported by patients, the estimated NRS-2002 score was automatically calculated through the algorithm set in the back end of R+ Dietitian and presented in the results interface. The algorithm was consistent with the scoring rules of the NRS-2002 [29].

##### Design and Nutritional Risk Score Estimation of the PG-SGA-SF Tool in R+ Dietitian

The PG-SGA-SF tool comprises the first 4 options of the Patient-Generated Subjective Global Assessment (PG-SGA), including weight history, food intake, symptoms, and activities and function, and is designed to be used by patients independently [31]. Studies have shown that inclusion of the first 4 options did not add further value as a screening tool compared with inclusion of the first 3 options [32]. Therefore, the PG-SGS-SF tool in R+ Dietitian only evaluates weight history, food intake, and symptoms.

Weight history was already evaluated by the question “Has your weight changed over the past three months?,” which was also used to evaluate the weight loss in the NRS-2002 tool. Food intake in the PG-SGS-SF tool was evaluated by the question “Has there been a reduction in your food intake recently?,” which was also used to evaluate the degree of food intake reduction in the NRS-2002 tool. Food intake was additionally evaluated by the question “What does your dietary look like recently?,” with the following 4 options: “fasting,” “liquid diet,” “soft food,” and “normal food.” Besides, a picture showing what the food looks like was presented next to the latter 3 options to help patients make choice. Symptoms were evaluated by the question “Have you had a bad appetite lately?” with 3 options, “never,” “occasionally,” and “frequently.”

Based on weight history, food intake, and symptoms reported by patients, the estimated PG-SGA-SF score was automatically calculated through the algorithm set in the back end of R+ Dietitian and presented in the results interface. The algorithm was consistent with the scoring rules of the PG-SGS-SF tool.

#### Part 3: Dietary Energy and Protein Assessment

The 24-hour dietary recall (24HR) is a traditional method for assessing dietary nutrient intake and is widely used in nutrition research [33-35]. However, it requires patients to recall any food, beverages, and water that they consumed, as well as extensive training of the interviewer. These make it burdensome and time-consuming, limiting its utility in clinical practice. For describing the mean usual intake of target nutrients, a “short dietary screener” is feasible. Blalock and colleagues [36] developed a short instrument for assessing dietary intakes of calcium and vitamin D, which only includes 22 foods and beverages that are rich in these 2 nutrients. Similar short tools for assessing dietary intake of cholesterol [37] and saturated fat are also found in the literature [38]. In this mini program, we only wanted to estimate the dietary energy and protein intakes in patients, so, as for a quick assessment, patients’ recalling all the food they consumed is not necessary because some foods contribute less to the energy and protein intakes. Therefore, we shortened the 24HR method to make it suitable for self-reporting and easier to complete, thereby allowing the daily energy and protein intakes of patients to be estimated quickly.

The China Health and Nutrition Surveys (CHNS) revealed that the main sources of dietary energy and protein among Chinese residents were cereals and animal foods [39-41]. Rice is the primary cereal type in China and milk is an important source of protein. Therefore, we designed 3 questions to assess dietary energy and protein intakes, “How much rice have you recently consumed on a daily basis?,” “How much meat have you recently consumed on a daily basis?,” and “How much milk have you recently consumed on a daily basis?.” An adjustable ruler (unit: gram or milliliter) presented under the 3 questions allowed the patients to report the amount of rice/meat/milk they consumed.

Before this study began, we conducted a pilot nutrition survey using 3 days of 24HR data (3d-24HRs) among hospitalized patients. Data on foods and nutrients derived from the survey were used to create the algorithm for calculating energy and protein intakes in R+ Dietitian. Based on the rice/meat/milk consumption reported, the estimated dietary energy and protein intakes were automatically calculated through the algorithm set in the back end of R+ Dietitian. Besides, the patients’ requirements for energy and protein were automatically estimated by the algorithm, which was based on the guideline from the CSPEN [11]. The difference between the estimation of dietary intakes of energy and protein and patients’ requirements was calculated automatedly and was presented on the results screen.

### Validity Evaluation of R+ Dietitian

#### Study Design, Setting, and Participants

This was a prospective diagnostic accuracy study conducted at the Oncology Department of Sichuan People’s Hospital, Chengdu, China, from March 2021 to April 2021. Eligible patients were adults aged 18-80 years with pathologically confirmed tumors who were able to communicate normally. We excluded patients with mental or psychological disorders, those with incomplete data, and those who were unwilling or unable to provide written informed consent. All patients registering to the Oncology Department in March 2021 and April 2021 were evaluated for study eligibility by a researcher, and consequently, this was a convenience sample of oncology patients.

#### Ethics Approval

This study was performed according to the Declaration of Helsinki and was approved by the Medical Ethics Committee of Sichuan Provincial People’s Hospital (2019/243). Written informed consent was obtained from all participants.

#### Test Methods

##### Index Test

Two dietitians (HX and QZ) were trained to use the R+ Dietitian program before the study started so that they can assist participants in using R+ Dietitian when needed. A clinician in the Oncology Department identified patients that met the inclusion and exclusion criteria and then informed 1 of the 2 dietitians about their eligibility.

On the day of hospital admission, eligible participants were asked by a dietitian (HX or QZ) to use R+ Dietitian for nutritional risk screening and dietary assessment of energy and protein. Participants or their family members used smartphones to open the WeChat app and then scanned a QR code to run the R+ Dietitian mini program. Registering in R+ Dietitian was not mandatory, so participants can use the mini program directly. The 3 parts of R+ Dietitian were completed by participants one by one. Estimations of the NRS-2002 score, the PG-SGA-SF score, and dietary energy and protein intakes were immediately presented on the results interface of R+ Dietitian once participants completed the program.

##### Reference Test

We used face-to-face interviews as the reference method. On the day of hospital admission, after patients self-evaluated R+ Dietitian, paper-based NRS-2002, PG-SGA-SF, and 3d-24HRs tools were administered according to the standard procedure by 2 trained dietitians (HX and QZ). 3d-24HRs collected data on participants’ food intake in the 3 days before the day of hospital admission. Intake of all foods consumed by participants in the 3 days and corresponding cooking methods, including stir-fry, braising, stew, etc., were recorded. If the food was a composite dish, its composition and the corresponding proportion would be further asked and recorded. To ensure the precision of intake recalled by participants, dietitians showed pictures of standard cutlery to the participants to help them assess the intake when 3d-24HRs were performed.

#### Data Collection

The baseline demographic and medical characteristics of the participants, including age, sex, means of paying medical costs, occupation, marital status, residence, education level, chronic diseases, cancer type, and family history of cancer, were obtained from the electronic medical records by a researcher (JZ).

Estimations of the NRS-2002 score, the PG-SGA-SF score, and dietary energy and protein intakes in R+ Dietitian were retrieved from the back end of R+ Dietitian by another researcher (CH). The NRS-2002 and PG-SGA-SF scores of the reference method were calculated according to the scoring rules of the 2 questionnaires and then entered into EpiData (EpiData Association) by the 2 dietitians (HX and QZ).

Dietary data obtained from 3d-24HRs were managed by the 2 dietitians. First, the individual food and the corresponding specific amount consumed by the participants each day were entered into MS Excel (Microsoft Corporation). Next, the raw weight of every individual food was calculated based on the raw-to-cook ratio of the food. Then, if the dish was cooked with oil, the approximate amount of the oil consumed was estimated based on the intake of the dish of the participants. Finally, the energy and protein intakes from each individual food were calculated based on China Food Composition Tables. The total energy and protein intakes each day were then calculated. The final estimated daily dietary energy and protein intakes was the mean of 3-day intake and was entered into EpiData.

#### Data Assessment and Statistical Analysis

The NRS-2002 scores of 3 or above identified patients at nutritional risk [29], whereas the PG-SGA-SF scores of 4 or above identified patients at nutritional risk [27,42]. The difference in dietary energy and protein intakes estimated through R+ Dietitian from those measured by dietitians was calculated.

All statistical analyses were performed using SAS 9.4 software (SAS Institute Inc.). First, continuous variables were analyzed for normality using the Kolmogorov–Smirnov test. Data were then described as mean (SD) or median (IQR). The Student *t* test or the rank sum test was applied accordingly. Categorical variables were described as frequencies and percentages, and then the *χ*^2^ test was applied. Accuracy, sensitivity, specificity, positive predictive value, negative predictive value (NPV), and κ values of the NRS-2002 and PG-SGA-SF tools in R+ Dietitian were calculated using the McNemar test for correlated proportions, respectively. The consistency of R+ Dietitian for dietary assessment with 3d-24HRs was tested using the Pearson or Spearman correlation test. All tests were 2-sided, and a significant level of 5% (*P*˂.05) was applied.

The validity of R+ Dietitian was evaluated using the cutoffs recommended by van Bokhorst–de van der Schueren et al [43], which were based on sensitivity and specificity. “Good” indicated both sensitivity and specificity exceeding 80%; “fair” indicated sensitivity or specificity exceeding 50% but lower than 80%; and “poor” indicated sensitivity or specificity lower than 50%. For other validation indices, the cutoffs of the correlation coefficients (CCs) followed Guilford’s [44] description as follows: “good,” CC ≥0.75; “fair,” CC ≥0.4 and <0.75; and “poor,” CC <0.4. The cutoffs for good, fair, and poor κ values [45] were ≥0.6, ≥0.4 and <0.6, and <0.4, respectively.

## Results

### Participants

**Figure 3:**
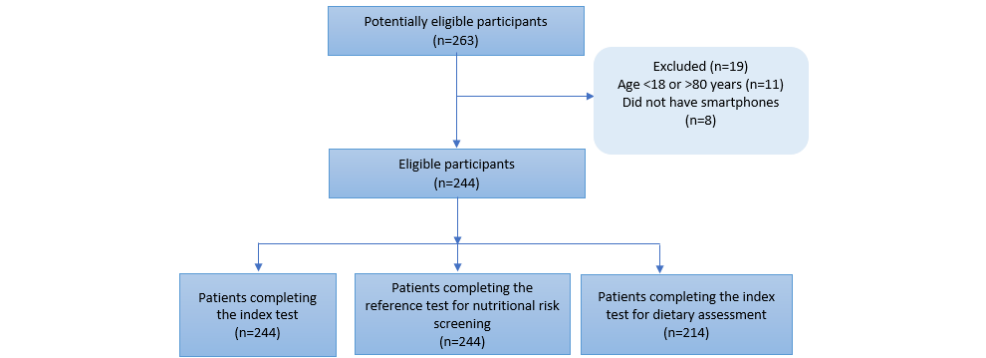
Flow of participants.

Figure 3 illustrates the flow of participants through the study. From March 2021 to April 2021, 263 patients were assessed for eligibility and were invited to participate in this study. Patients who were excluded or who withdrew from this study and the corresponding reasons were noted. Overall, 244 patients were recruited to evaluate the validity of R+ Dietitian for nutritional risk screening, and 214 patients were included to evaluate the validity of R+ Dietitian for dietary intake assessment.

### Characteristics of Participants

The baseline characteristics of the participants included in this study are presented in Table 1. The median (IQR) age of participants was 59 (51–68) years and the mean (SD) weight and BMI were 58.6 (8.9) kg and 22.2 (2.9) kg/m^2^, respectively. Most participants were male (156/244, 63.9%). The most common diagnosis was gastrointestinal tumor (107/244, 43.9%) and the least common diagnosis was head and neck cancer (17/244, 6.9%).

**Table 1:** Baseline characteristics of participants (N=244).

Variables		Values
Age (years), median (IQR)		59 (51-68)
Weight (kg), mean (SD)		58.6 (8.9)
BMI (kg/m^2^), mean (SD)		22.2 (2.9)
**Gender, n (%)**		
	Male	156 (63.9)
	Female	88 (36.1)
**Means of paying medical costs, n (%)**		
	Social security	230 (94.3)
	Self-paid	14 (5.7)
**Occupation, n (%)**		
	Famer/worker	27 (11.1)
	Retirement	45 (18.4)
	Other	172 (70.5)
**Marital status, n (%)**		
	Married	241 (98.8)
	Unmarried	3 (1.2)
**Residence, n (%)**		
	Rural	130 (53.3)
	City	114 (46.7)
**Education, n (%)**		
	Primary	28 (11.5)
	Secondary	197 (80.7)
	Senior	19 (7.8)
**Hypertension, n (%)**		
	Yes	40 (16.4)
	No	204 (83.6)
**Diabetes mellitus, n (%)**		
	Yes	22 (9)
	No	222 (91)
**Cancer family history, n (%)**		
	Yes	16 (6.6)
	No	228 (93.4)
**Cancer type, n (%)**		
	Head and neck tumor	17 (6.9)
	Gastrointestinal tumor	107 (43.9)
	Respiratory tumor	76 (31.1)
	Other	44 (18)

### Agreement Between the NRS-2002 Tool in R+ Dietitian and the NRS-2002 Tool Administered by Dietitians

Table 2 presents the screening results of the NRS-2002 tool in R+ Dietitian in comparison to dietitians’ screening. According to the NRS-2002 tool in R+ Dietitian, 33.2% (81/244) of patients were at nutritional risk, which was significantly higher than in the screening by dietitians (42/244, 17.2%; *P*<.001; McNemar test). The NRS-2002 tool in R+ Dietitian was in fair agreement with the NRS-2002 tool administered by dietitians (Table 3; both sensitivity and specificity >50%, κ>0.42, and CC >0.4). In addition, self-screening by patients using the NRS-2002 tool in R+ Dietitian had a high NPV (95.1%), which indicates that self-screening by patients using the NRS-2002 in R+ Dietitian can strongly predict those that are not at nutritional risk.

**Table 2:** Cross tabulation of nutritional risk according to the NRS-2002^a^ tool in R+ Dietitian and the NRS-2002 tool administered by dietitians.

R+ Dietitian (patients’ self-screening)	Dietitians’ screening		Total (N=244)
	Positive (n=42)	Negative (n=202)	
Positive, n (%)	34 (13.9)	47 (19.3)	81 (33.2)
Negative, n (%)	8 (3.3)	155 (63.5)	163 (66.8)
Total (n=244)	42 (17.2)	202 (82.8)	244 (100)

^a^Nutritional Risk Screening.

**Table 3:** Sensitivity, specificity, positive predictive value, negative predictive value, κ value, and correlation coefficient of patients’ self-screening using the NRS-2002 tool in R+ Dietitian.

Index	NRS-2002^a^ in R+ Dietitian (patients’ self-screening), % (95% CI)	*P* value
Accuracy	77.5 (71.7-82.5)	N/A^b^
Sensitivity	81.0 (65.4-90.9)	N/A
Specificity	76.7 (70.2-82.2)	N/A
Positive predictive value	42.0 (31.3-53.5)	N/A
Negative predictive value	95.1 (90.2-97.7)	N/A
κ value	0.42 (0.30-0.54)	N/A
Correlation coefficient	0.62 (0.54-0.70)	<.001

^a^Nutritional Risk Screening.

^b^Not applicable.

### Agreement Between the PG-SGA-SF Tools in R+ Dietitian and the PG-SGA-SF Tool Administered by Dietitians

Table 4 shows the screening results of the PG-SGS-SF tool in R+ Dietitian in comparison to dietitians’ screening. According to the PG-SGS-SF tool in R+ Dietitian, 47.1% (115/244) of patients were at nutritional risk, which was significantly higher than in the screening by dietitians (58/244, 23.8%; *P*<.001) according to the McNemar test. Further, the PG-SGS-SF tool in R+ Dietitian was fairly in agreement with the NRS-2002 tool administered by dietitians (Table 5; both sensitivity and specificity >50% and CC >0.4). Besides, as with the NRS-2002 tool in R+ Dietitian, patients’ self-screening using the PG-SGS-SF tool in R+ Dietitian had a high NPV (95.1%).

**Table 4:** Cross tabulation of nutritional risk according to the PG-SGA-SF^a^ tool in R+ Dietitian and the PG-SGS-SF tool administered by dietitians.

R+ Dietitian (patients’ self-screening)	Dietitians’ screening, n (%)		
	Positive (n=58)	Negative (n=186)	Total (N=244)
Positive, n (%)	49 (20.1)	66 (27.0)	115 (47.1)
Negative, n (%)	9 (3.7)	120 (49.2)	129 (52.9)
Total (n=244)	58 (23.8)	186 (76.2)	244 (100)

^a^Patient-Generated Subjective Global Assessment Short Form.

**Table 5:** Sensitivity, specificity, positive predictive value, negative predictive value, κ value, and correlation coefficient of patients’ self-screening using the PG-SGA-SF^a^ tool in R+ Dietitian.

Index	PG-SGA-SF in R+ Dietitian (patients’ self-screening), % (95% CI)	*P* value
Accuracy	69.3 (63.1-75.0)	N/A^b^
Sensitivity	84.5 (72.1-92.2)	N/A
Specificity	64.5 (57.1-71.3)	N/A
Positive predictive value	42.6 (33.5-52.2)	N/A
Negative predictive value	93.0 (86.8-96.6)	N/A
κ value	0.37 (0.26-0.47)	N/A
Correlation coefficient	0.56 (0.47-0.64)	<.001

^a^Patient-Generated Subjective Global Assessment Short Form.

^b^Not applicable.

### Agreement Between Dietary Energy and Protein Intake Assessment in R+ Dietitian and Assessment Performed by Dietitians

The dietary energy and protein intakes estimated by R+ Dietitian and dietitians are presented in Table 6. Both the estimated energy and protein intakes were significantly higher (*P*<.001 for both) in R+ Dietitian, compared with the 3d-24HRs. Estimations of energy and protein intakes in R+ Dietitian were 10.7% and 29.0% higher than those in 3d-24HRs, respectively (Table 7). Nevertheless, R+ Dietitian was still in moderate agreement with 3d-24HRs for both energy and protein intake assessment (Table 7; both CC >0.4).

**Table 6:** Estimation of energy and protein intakes in R+ Dietitian compared with the 3d-24HRs administered by dietitians.

Dietary intake	R+ Dietitian	3d-24HRs^a^	*P* value
Energy (kcal), mean (SD)	1578.3 (468.4)	1434.1 (528.8)	<.001
Protein (g), median (IQR)	79.0 (62.7-95.3)	61.7 (43.0-82.8)	<.001

^a^Three days of 24-hour dietary recall.

**Table 7:** Correlation coefficient and absolute and relative differences of dietary energy and protein intake estimation in R+ Dietitian against 3d-24HRs^a^.

Index	Energy	*P* value	Protein	*P* value
Correlation coefficient (95% CI)	0.59 (0.49-0.67)	<.001	0.47 (0.36-0.57)	<.001
Absolute difference (kcal or g), mean (SD)	144.2 (454.8)	<.001	14.7 (29.2)	<.001
Relative difference (%), median (IQR)	10.7 (9.5-39.8)	<.001	29 (3.1-68.1)	<.001

^a^Three days of 24-hour dietary recall.

## Discussion

### Principal Findings

The R+ Dietitian mini program is developed for use among hospitalized patients with cancer. This prospective study demonstrates the validity of R+ Dietitian for nutritional risk screening and dietary energy and protein intake assessment in oncology department. Overall, the NRS-2002, PG-SGA-SF, and dietary energy and protein intake assessments in R+ Dietitian were in fair agreement with those performed by trained dietitians, although the number of patients identified at nutritional risk was more and the estimated dietary energy and protein intakes were higher in R+ Dietitian, compared with the reference methods. Both the NRS-2002 and the PG-SGA-SF tools in R+ Dietitian showed an excellent ability to predict those who were not at nutritional risk.

To the best of our knowledge, no similar mini program has been developed and this is the first prospective study to examine the validity of a digital smartphone-based self-administered tool for nutritional risk screening and dietary assessment in the oncology department.

### The Validity of R+ Dietitian’s Nutritional Risk Screening

For nutritional risk screening in hospitals, the NRS-2002 is recommended by the CSPEN [11] and the PG-SGA-SF is widely used among patients with cancer [26-28]. Therefore, we developed nutritional risk screening in R+ Dietitian based on both the NRS-2002 and the PG-SGA-SF. Our study shows that nutritional risk evaluated by the NRS-2002 and PG-SGA-SF tools in R+ Dietitian had fair validity.

No similar smartphone-based app for nutritional risk self-screening has been developed, although a computer-based self-screening app is available [21,46]. This app is the electronic version of the MUST and was developed for hospital outpatients. It showed high agreement with the MUST administered by health care professionals (κ=0.74-1.00). These values are higher than the NRS-2002 and PG-SGA-SF tools in R+ Dietitian. The different degrees of agreement between the electronic version of the MUST and R+ Dietitian can be explained as follows: First, in the study of the electronic version of the MUST, the weight and height were measured using a weighing scale and a portable height measure, respectively, in the outpatients’ waiting room by patients. Consequently, the weight and height reported by patients are more likely to be objective than those in R+ Dietitian. Both dietitians in our study and health care professionals in the study of the electronic version of the MUST used weight and height scales to measure the weight and height, respectively, of participants. However, there was a higher deviation in the weight and height reported by patients and professionals in our study, compared with those in the study of the electronic version of the MUST. BMI calculated based on weight and height is a factor for nutritional risk scoring in both R+ Dietitian and the electronic version of the MUST. Weight was also used to calculate the degree of weight loss in the past 3-6 months, which is also a criterion for evaluating nutritional risk. Hence, the different methods applied to measure weight and height by participants may be one of the reasons for the difference in agreement between these 2 studies. However, bias induced from recall is common in surveys [47-51] and can hardly be avoided. Second, the NRS-2002 tool in R+ Dietitian additionally evaluates the degree of food intake reduction using the question, “Has there been a reduction in your food intake recently?” with 3 options, “slight reduction (reduction of about 25%-50%),” “moderate reduction (reduction of about 50%-75%),” and “severe reduction (reduction of about 75%-100%).” For this question, some patients may compare their recent food intake with food intake several weeks ago rather than their normal requirement of food intake, whereas dietitians compared patients’ recent food intake with their normal requirement. Third, the PG-SGS-SF tool in R+ Dietitian did not evaluate the symptoms affecting patients’ nutritional intake, but dietitians did. The electronic version of the MUST was developed for hospital outpatients and requires patients to use the weight scale and the portable height measure tool, which is less feasible for inpatients, as they are busy with completing their admission procedures while being admitted to the hospital. In addition, the electronic version of the MUST was computer based, which makes it less flexible compared with R+ Dietitian, which is a smartphone-based app for self-screening.

Nutritional risk screening in R+ Dietitian showed a fair agreement with the dietitians’ screening. As the first smartphone-based tool for nutritional risk self-screening, it displayed potential ability to be used in clinical practice for nutritional risk screening.

### The Validity of R+ Dietitian’s Dietary Energy and Protein Intake Assessment

The energy intake was overestimated by 144 kcal/day in R+ Dietitian compared with the estimation from 3d-24HRs performed by dietitians. Our finding is different from other self-administered dietary assessment apps. Compared with 24HRs or the Food Frequency Questionnaire (FFQ), most of these apps underestimated energy intake from –8 to –466 kcal/day [24,52-60], and 1 app overestimated energy intake by 55 kcal/day [17]. The absolute mean difference of estimated energy intake between R+ Dietitian and 3d-24HRs was higher in our study compared with the absolute difference in 5 studies (from 8.1 to 101 kcal/day) [24,55-57,60] but was lower compared with the absolute difference reported in 6 studies (from 145.1 to 466.0 kcal/day) [22,52-54,58,59]. The following might have contributed to the difference between R+ Dietitian and other apps. First, all the other validity studies required participants to use the experimental app to record their food intake for several days [17,23,24,53,54,56,57,59,60] or even few months [52,58]. Based on the data inputted, the daily energy intake and the mean energy intake over the study period would be calculated automatically by the app. By contrast, in our study, patients self-reported their mean food intake but the mean energy intake was calculated automatically by R+ Dietitian. The different methods of collecting data on food intake between R+ Dietitian and other tools may result in differences in the energy intake estimated. Second, other apps recorded all the foods consumed by participants, whereas only 3 food groups were reported by patients in R+ Dietitian. R+ Dietitian was developed to be a digital tool that can quickly assess energy and protein intakes of patients, thereby allowing clinicians, who generally have limited time to analyze these, to have an approximate overview of the intake level of the 2 nutrients (ie, energy and protein). Further and more complex and detailed dietary assessment is needed if patients are at nutritional risk. Third, patients in most validity studies for self-administered dietary assessment apps were young. For example, their sample population was recruited in high school [52] or in a university setting [24,53,55-57,59]. The median age of participants in our study was 59 years, which is much higher than that in these studies. Young and old people may have different tendencies in self-reporting studies. For the 2 studies that recruited old people or hospitalized patients [17,60], the absolute mean difference in estimated energy intake between the experimental app and the reference method was lower (55 kcal/day and 101 kcal/day, respectively), but in another study [58] this was higher (408.8 kcal/day) than that in our study (144 kcal/day).

The estimation of protein intake in R+ Dietitian was higher than that from 3d-24HRs by 14.7 g/day. Mescoloto and colleagues [61] and Bucher Della Torre and coworkers [60] also reported overestimation of protein intake in 2 digital dietary recording apps by 2.1 and 2.0 g/day, respectively, compared with measured energy expenditure and 2 unannounced 24-h phone dietary recalls on overlapping days. In contrast to our study and these 2 studies [60,61], 7 studies found an underestimation of protein intake in phone-based dietary recording apps from –2.6 to –28 g/day [17,24,52,53,55,56,58], compared with 24HRs or FFQs or paper-based food records. The absolute difference between the experimental app and the reference method in our study was higher than that in 4 studies (from –2.6 to –10.5 g/day) [17,24,55,56], but was lower than that in 3 other studies (from –21.5 to 28.0 g/day) [52,53,58]. For the estimation of protein intake, just like the estimation of energy intake, other studies asked participants to use the dietary recording apps to record all the foods they consumed during the study period in real time, whereas we asked participants in our study to recall their intake of 3 recent food groups for a quick estimation during their hospital admission. In general, real-time recording may be more precise than recall in dietary assessment [62]. But still, the difference in the estimation of protein intake between R+ Dietitian and 3d-24HRs was lower than that between 3 dietary recording apps and 24HRs or paper-based food records [52,53,55].

Nowadays, similar to traditional dietary intake assessment tools, including 24HRs, FFQs, and weighed or nonweighed food records, all smartphone-based digital dietary intake assessing apps require users to report on all the foods or food groups they consume [17,22-24,52-60,63]. However, this approach may not be suitable for all hospitalized patients. On the one hand, these apps require users to record their food intake for several days, or even few months, which cannot help clinicians to understand their patients’ nutritional intake within the short period available for initial assessment. The only 1 study that recruited hospitalized patients [17] asked them to use the experimental app to record food intake for 2 days; however, MyFood was developed for inpatients with nutritional risk rather than all hospitalized patients, which may limit its usage in other patient groups. In this case, the nutritional intake of other nonnutritional-risk patients can be missed by the clinicians. On the other hand, recording all foods or food groups is also time-consuming and burdensome for patients, so some may forget to record their food intake at times. R+ Dietitian was used on the day of hospital admission and only includes 3 food groups, reducing patients’ burden and helping clinicians to have a quick and approximate overview of the patients’ energy and protein intakes. If the patients are diagnosed to have nutritional risk, further comprehensive dietary assessments are needed.

### Strengths and Limitations

To our knowledge, R+ Dietitian is the first digital smartphone-based self-administered tool for both nutritional risk screening and dietary assessment for hospitalized patients with cancer. This is a strong strength of this study. Another strength is that R+ Dietitian was developed based on the NRS-2002 and PG-SGA-SF tools. The NRS-2002 is the only tool validated by a retrospective analysis of 128 randomized controlled trials [29] and is recommended by several professional communities [11,64]. The PG-SGS-SF is specific for patients with cancer and has been proved to be an effective tool to screen nutritional risk for patients with cancer [28,31,42,65,66]. Experienced dietitians, nurses, and patients were involved in the development of R+ Dietitian, which is also an important strength. R+ Dietitian was developed as a subapp within the WeChat ecosystem, which is China’s most popular messaging app with a monthly user base of more than 1 billion people [25]. This makes it commonly available and easily accessible. All participants in our study had installed WeChat on their smartphones and thus the need to download a new app was not required, thereby saving their time.

A limitation of this study is that we only recruited hospitalized patients with cancer in 1 hospital, which may limit the generalizability of our findings. However, R+ Dietitian was developed based on the NRS-2002 tool, which has been used among various types of patients. Hence, we propose that R+ Dietitian can also be used for other types of patients, but this needs further research. Besides, usability is one of the important factors determining the tool’s actual usefulness in practical settings [67]. The usability of R+ Dietitian was not evaluated in this study. The 2 dietitians responsible for asking the patients to use R+ Dietitian reported that none of the patients reported difficulties while using the tool.

### R+ Dietitian’s Potential for Nutritional Risk Screening and Dietary Intake Assessment Among Hospitalized Patients With Cancer

Based on the findings in this study, we propose that R+ Dietitian has a large potential to be a tool for nutritional risk screening and dietary intake assessment among hospitalized patients with cancer. R+ Dietitian may provide support for nurses and clinicians to perform nutritional risk screening and dietary assessment among hospitalized patients with cancer, enhancing the rate and efficiency of nutritional risk screening and dietary assessment among this patient group. In addition, R+ Dietitian is a WeChat-based tool, which makes it commonly available and may potentially increase its use among hospitalized patients. Further validity of this study for other types of patients may be helpful to expand the underlying use of R+ Dietitian in hospital settings.

### Conclusions

We have developed a digital smartphone-based self-administered instrument for nutritional risk screening and dietary assessment among hospitalized patients with canner. The instrument enables the evaluation of estimated dietary intake of energy and protein against individual’s requirements. The identified nutritional risk and assessment of dietary energy and protein intakes in R+ Dietitian displayed a fair agreement with the screening and assessment conducted by dietitians. R+ Dietitian has the potential to be a tool for nutritional risk screening and dietary intake assessment among hospitalized patients with cancer.

## Abbreviations

3d-24HRs: 3 days of 24-hour dietary recall
24HR: 24-hour dietary recall
ASPEN: American Society for Parenteral and Enteral Nutrition
CC: correlation coefficient
CHNS: China Health and Nutrition Surveys
CSPEN: Chinese Society for Parenteral and Enteral Nutrition
ESPEN: European Society for Clinical Nutrition and Metabolism
FFQ: Food Frequency Questionnaire
MUST: Malnutrition Universal Screening Tool
NPV: negative predictive value
NRS-2002: Nutritional Risk Screening
PG-SGA-SF: Patient-Generated Subjective Global Assessment Short Form
